# Does Chronic Obstructive Pulmonary Disease with or without Type 2 Diabetes Mellitus Influence the Risk of Lung Cancer? Result from a Population-Based Cohort Study

**DOI:** 10.1371/journal.pone.0098290

**Published:** 2014-05-22

**Authors:** Te-Chun Shen, Wei-Sheng Chung, Cheng-Li Lin, Chang-Ching Wei, Chia-Hung Chen, Hung-Jen Chen, Chih-Yen Tu, Te-Chun Hsia, Chuen-Ming Shih, Wu-Huei Hsu, Chi-Jung Chung

**Affiliations:** 1 Division of Pulmonary and Critical Care Medicine, Department of Internal Medicine, China Medical University Hospital and China Medical University, Taichung, Taiwan; 2 Division of Pulmonary and Critical Care Medicine, Department of Internal Medicine, Chu Shang Show Chwan Hospital, Nantou, Taiwan; 3 Department of Internal Medicine, Taichung Hospital, Ministry of Health and Welfare, Taichung, Taiwan; 4 Department of Healthcare Administration, Central Taiwan University of Science and Technology, Taichung, Taiwan; 5 Department of Public Health, College of Public Health, China Medical University, Taichung, Taiwan; 6 Management Office for Health Data, China Medical University Hospital, Taichung, Taiwan; 7 Division of Nephrology, Department of Pediatrics, China Medical University Hospital and China Medical University, Taichung, Taiwan; 8 Department of Health Risk Management, College of Public Health, China Medical University, Taichung, Taiwan; 9 Department of Medical Research, China Medical University Hospital, Taichung, Taiwan; University Hospital Freiburg, Germany

## Abstract

**Background:**

Previous studies have suggested that chronic obstructive pulmonary disease (COPD) is an independent risk factor for lung cancer. There are some evidence that people with diabetes are at a risk of developing many forms of cancer, but inconclusive with regard to lung cancer. The aim of this study was to evaluate whether COPD with or without type 2 diabetes mellitus (T2DM) influences the risk of developing lung cancer.

**Methods:**

This is a retrospective cohort study consisting of 20,730 subjects newly diagnosed with COPD (“cases”). Their data was collected from the National Health Insurance system of Taiwan from 1998 to 2010. Among these patients, 5,820 patients had T2DM and 14,910 patients did not have T2DM. The retrospective matched control group consisted of 20,729 subjects without either COPD or T2DM. The control group was matched with the cases for sex, age, and index year (the year that the patient was diagnosed with COPD). The subjects were followed until the end of 2011.

**Results:**

The findings of our study showed that the risk of lung cancer was higher in the COPD group than in the non-COPD group, with adjusted hazard ratio (HR) of 5.02 [95% confidence interval (CI) = 4.23–5.94] among total case group, adjusted HR was 5.38 (95% CI = 4.52–6.40) in the cohort without T2DM and adjusted HR was 4.05 (95% CI = 3.26–5.03) in the cohort with T2DM. We observed a significantly protective effect from lung cancer (adjusted HR = 0.75, 95% CI = 0.63–0.90) of diabetic cohort than non-diabetic cohort among patients with COPD.

**Conclusion:**

Patients with COPD had a significantly higher risk of developing lung cancer than healthy people. However, there was a protective effect of T2DM for lung cancer among patients with COPD. Further investigation may be needed to corroborate the mechanism or bring up reliable reasons.

## Introduction

Lung cancer is the most common cancer in terms of both incidence and mortality worldwide. Over 1.38 million people die from lung cancer every year [1]. Cigarette smoking is the primary etiologic agent in 85–90% of all lung cancers [2], but only 10–15% of active smokers develop lung cancer [3]. In addition, lung cancer is the seventh most common cause of cancer deaths worldwide in never smokers [4]. The incidence and prevalence of lung cancer are both increasing. Chronic obstructive pulmonary disease (COPD) is known as a persistent airflow limitation that is usually progressive and associated with an enhanced chronic inflammatory response in the airways [5]. It is the fourth leading cause of death worldwide, with a reported prevalence rates between 5% and 13% [6]–[9]. Cigarette smoking is also the greatest risk factor for the development of COPD [10], [11].

COPD and lung cancer are two of the most important smoking-related diseases worldwide [12], [13]. Many consider the presence of COPD itself to be an independent risk factor for lung cancer whether its association with smoking or not [14], [15], but some others argue that they are just manifestations of the same exposure [16]. Wang *et al*. recently published a meta-analysis of the relationship between COPD and lung cancer and they concluded COPD was significantly associated with the odds for increased risk of lung cancer [pooled odds ratio (OR) = 2.76; 95% confidence interval (CI) = 1.85–4.11]; and these associations were more pronounced in smoker than in non-smokers [17]. However, there are inherent limitations to meta-analysis such as heterogeneity across studies, particularly in the types of controls used and in diagnostic methods.

Diabetes mellitus (DM) tremendously affects public health because of its high prevalence and complications. The worldwide prevalence of DM was 171 million persons in 2000 and reached 346 million in 2011 [18]. DM increases the risk of a wide range of vascular diseases [19]. Emerging evidence has shown that DM is associated with certain types of cancer, which is possible because the complex system of glucose metabolism triggers carcinogenesis when defects occur in some relevant processes [20], [21]. Although some studies have reported an increased risk of certain cancers in patients with DM, other studies that have found no significant increase or decrease in the risk of developing other type of cancers. A recent consensus review mentioned that the relative risks imparted by diabetes are greatest (approximately 2-fold or higher) for developing cancer of the liver, pancreas, and endometrium, and lesser (approximately 1.2- to 1.5-fold) for cancers of the colon/rectum, breast, and bladder. Other types of cancers (e.g. lung cancer) do not appear to be associated with an increased risk in diabetes and the evidence for others (e.g., kidney and non-Hodgkin lymphoma) is inconclusive. However, there are few studies to date have explored links with type 1 diabetes [22].

At present, it is still unclear whether COPD with or without type 2 DM (T2DM) influences the risk for lung cancer either globally or in Taiwan. The purpose of this study was to evaluate COPD patients with or without T2DM and the subsequent risk of lung cancer by conducting a population-based cohort study from the National Health Insurance Research Database (NHIRD) in Taiwan. The National Health Insurance (NHI) system of Taiwan provided a nationwide, large-scale cohort dataset, which has been used for various studies over several years [23]–[25]. To the best of our knowledge, this is the first nationwide population-based study evaluating the relationships among COPD, T2DM, and lung cancer risk.

## Materials and Methods

### Data Source

This study was exempted from full ethical review by the International Review Board, China Medical University and Hospital Research Ethics Committee (IRB permit number: CMU-REC-101–012). Department of Health of Taiwan established the NHI program in 1995, by incorporating 13 public insurance programs with 99% of 23.7 million of population covered in the system by 1998 [26]. The National Health Research Institute (NHRI) is responsible for managing the claims data and also established the Taiwan National Health Insurance Research Database (TNHIRD) for public use. In this study, we obtained a subset of the Longitudinal Health Insurance Database 2000 (LHID2000), from NHRI. The LHID2000 contains medical claims data of 1 million individuals randomly selected from all insured people registered from 1996 to 2011. We used the scrambled identification numbers to link data sets to safeguard the confidentiality of the insured population without ethical violation. The diagnostic codes were recorded according to International Classification of Diseases, Ninth Revision (ICD-9-CM) coding standards.

### Sampled Participants

Using claims data from 1998 to 2010, we identified patients with newly diagnosis COPD (ICD-9-CM 491, 492, and 496). Patients with both COPD and T2DM (ICD-9-CM 250) were selected as T2DM cohort and COPD patients without T2DM (free of T2DM diagnosis until the end of follow-up) as non-DM cohort. The date of diagnosis of COPD was defined as the “index date.” Patients were excluded if they were younger than 20 years or had a history of lung cancer (ICD-9-CM 162) prior to the index date.

From all NHI beneficiaries, a 1∶1 control cohort, matched for gender, age within 5 years, and index year was randomly selected among patients without COPD and T2DM. We also incorporated inpatient and outpatient diagnosis records to ascertain the baseline comorbidities, including pneumoconiosis (ICD-9-CM 500, 502, 503, 504, 505), interstitial lung disease (ICD-9-CM 516), pulmonary tuberculosis (TB) (ICD-9-CM 010, 011, 012, 018).

### Main Outcome

Newly diagnosed patients with lung cancer were confirmed by the Registry for Catastrophic Illness Patient Database (RCIPD), which is a subset of data from the TNHIRD. Lung cancer is categorized as a catastrophic illness in the NHI system of Taiwan. The patients who are newly diagnosed with lung cancer can apply for a “catastrophic illness certification” which is issued by the government. Issuing a catastrophic illness certification is a careful process, which involves review of medical records, images, and/or pathology reports by a panel of specialists and experts on this disease. Each patient was followed until a diagnosis of lung cancer was confirmed or until the patient was lost to follow up, died, withdrew from the insurance system, or reached the end of the follow-up period (December 31, 2011).

### Statistical analysis

We compared the distribution of sociodemographic factors (age, sex, urbanization level, monthly income) and the proportions of comorbidities between the cohorts with and without COPD by Chi-square-test and *t*-test. The incidence of lung cancer in the three cohorts of patients (COPD without T2DM, COPD with T2DM, and without COPD/T2DM) was calculated in the follow-up period until the end of 2011. Univariate analysis and multivariable Cox proportional hazard models were used to estimate the hazard ratios (HRs) and 95% CIs for the risk of developing lung cancer. The multivariate models were adjusted for age, sex, urbanization level, monthly income, and comorbidities of pneumoconiosis, interstitial lung disease, pulmonary TB. We used the Kaplan–Meier analysis and the log-rank test to examine the statistical significance of the differences among the three cohorts. All data analyses were performed by SAS statistical software for windows (Version 9.1; SAS Institute, Inc., Cary, NC, USA), and the significance level was set to be 0.05.

## Results

In total, we included 20,730 patients with COPD (14,910 subjects without T2DM and 5,820 subjects with T2DM). The control cohort consisted of 20,729 subjects. The baseline characteristics of the patients in the three cohorts are presented in Table 1. Compared to the control cohort (mean age 65 years), the COPD without T2DM cohort had a similar mean age of 64.8 years and the COPD with T2DM cohort had a significantly higher mean age of 68.9 years. Among the three cohorts, the majority of patients were male (66.9% in COPD without T2DM, 57.1% in COPD with T2DM, and 64.1% in control, respectively). Compared to control cohort, patients with COPD tended to live in lower urbanized levels and had lower income. Patients with COPD also had a higher prevalence of all comorbidities than that of the control cohort. The mean duration of follow-up for control cohort was 8.24 years, approximately 1 year longer than that for COPD without T2DM (7.42 years), and COPD with T2DM (7.01 years).

**Table 1:** Comparison of demographics and comorbidity between patients with COPD and controls.

			COPD						
	Control (N = 20729)		Without T2DM (N = 14910)		With T2DM (N = 5820)		Total (N = 20730)		
	n	%	n	%	n	%	n	%	p-value
Age (years)									0.99
<65	8185	39.5	6301	42.3	1884	32.4	8185	39.5	
65–74	6629	32.0	4427	29.7	2202	37.8	6629	32.0	
≥75	5915	28.5	4182	28.1	1734	29.8	5916	28.5	
Mean (SD) ^#^	65.0	14.6	64.8	15.5	68.9	10.8	65.9	14.5	<0.0001
Sex									0.99
Female	7439	35.9	4942	33.2	2497	42.9	7439	35.9	
Male	13290	64.1	9968	66.9	3323	57.1	13291	64.1	
Urbanization level†									<0.0001
1 (highest)	5611	27.1	3607	24.2	1371	23.6	4978	24.0	
2	5370	25.9	3901	26.2	1573	27.0	5474	26.4	
3	3568	17.2	2583	17.3	926	15.9	3509	16.9	
4 (lowest)	6180	29.8	4819	32.3	1950	33.5	6769	32.7	
Monthly Income (NTD^&^)									<0.0001
<15,000	7259	35.0	5216	35.0	2222	38.2	7438	35.9	
15,000–19,999	9304	44.9	7076	47.5	2727	46.9	9803	47.3	
≥20,000	4166	20.1	2618	17.6	871	15.0	3489	16.8	
Comorbidity									
Pneumoconiosis	141	0.68	282	1.89	82	1.41	364	1.76	<0.0001
Interstitial lung disease^‡^	5	0.02	11	0.07	8	0.14	19	0.09	<0.0001
Pulmonary TB	339	1.64	968	6.49	362	6.22	1330	6.42	<0.0001

Chi-square test compared to total COPD; ^#^:Two sample t-test; ^‡^:Fisher exact test

† The urbanization level was categorized by the population density of the residential area into 4 levels, with level 1 as the most urbanized and level 4 as the least urbanized. ^&^NTD: New Taiwan dollar.

**Figure 1:**
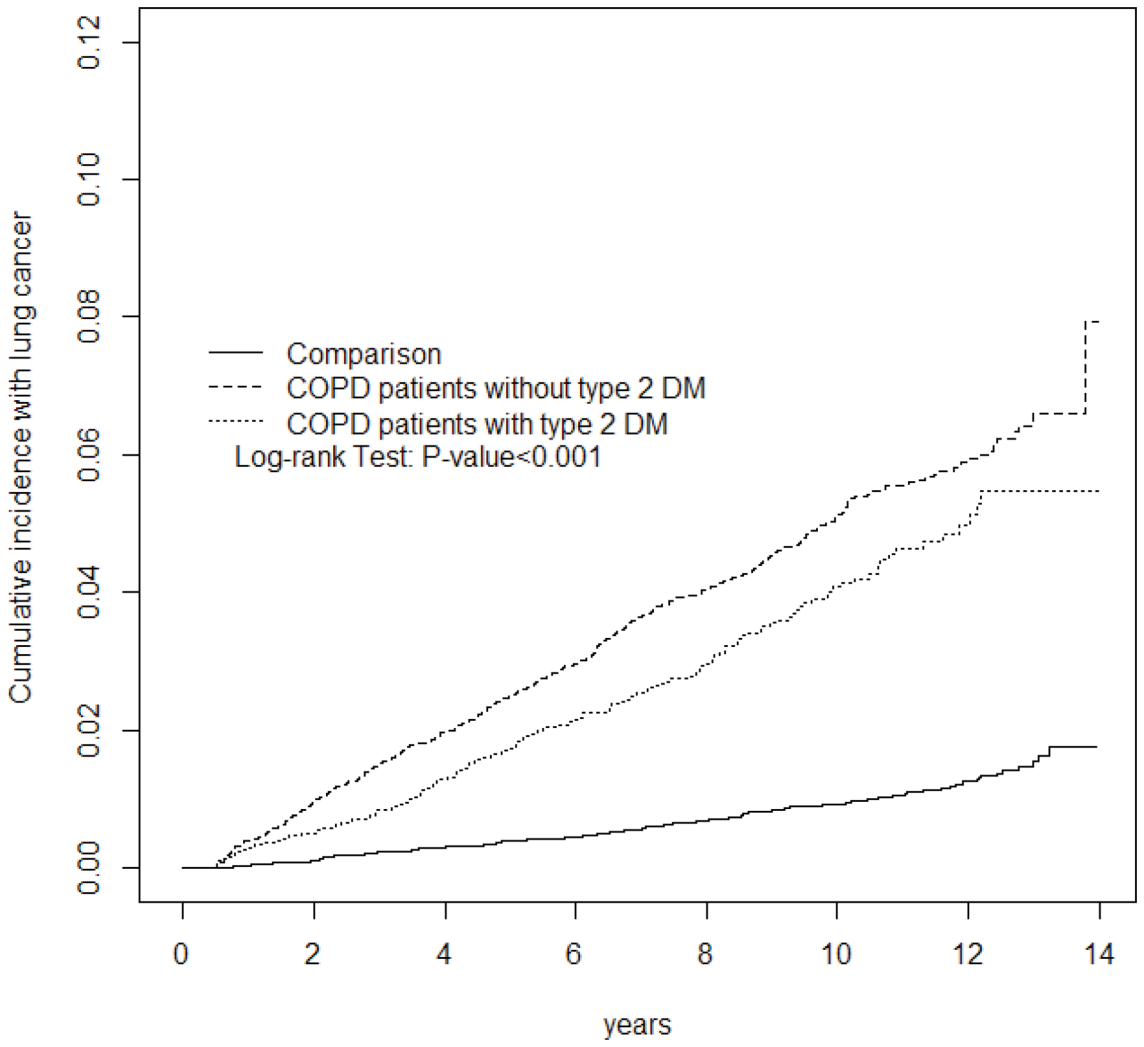
Cummulative incidence of lung cancer compared among COPD patients with T2DM, without T2DM and comparison subjects.

As shown in figure 1, the cumulative incidence of lung cancer estimated by Kaplan–Meier analysis revealed significant differences among the three cohorts over follow-up period (p<0.001). The cumulative incidence rate was much higher in COPD patients without T2DM than in those in the COPD with T2DM and control cohort (p = 0.0036 and p<0.001, respectively). In addition, the higher rate also observed in the COPD with T2DM than control cohort (p<0.001).

Following a 14-year follow-up, a high incidence rate of lung cancer was observed in the COPD cohorts without and with T2DM compared with the control cohort (5.13, 3.94, and 0.98, respectively). Furthermore, the total COPD cohort (adjusted HR = 5.02, 95% CI = 4.23–5.94), the COPD without T2DM cohort (adjusted HR = 5.38, 95% CI = 4.52–6.40), and COPD with T2DM cohort (adjusted HR = 4.05, 95% CI = 3.26–5.03) were all associated with a significantly higher risk of developing lung cancer (Table 2). The incidence of lung cancer was greater in men than in women in all three cohorts. By age stratification, the highest incidence rate of lung cancer was observed for the age group of 65–74 years in the COPD cohorts without and with T2DM compared with the control cohort (8.02, 5.18, and 1.32, respectively). However, the risk of lung cancer was highest in COPD patients without T2DM aged ≤64 years (adjusted HR  = 6.74, 95% CI = 4.63–9.81) and in COPD patients with T2DM aged ≤64 years (adjusted HR = 6.00, 95% CI = 3.76–9.56) compared with the control cohort after adjusting for risk factors. Regarding the highest urbanization level or high monthly income variables, COPD patients without T2DM had a significantly greater risk of developing lung cancer compared with the control cohort. This association was similar for the risk of lung cancer among the COPD with T2DM cohort.

**Table 2:** Hazard ratios of lung cancer between COPD without T2DM and control subjects as well as COPD with T2DM and control subjects stratified by demographics and comorbidity.

.	Control (N = 20729)		Without T2DM (N = 14910)		Crude HR * (95% CI)	Adjusted HR^†^	With T2DM (N = 5820)		Crude HR* (95% CI)	Adjusted HR^†^
	Case	Rate^#^	Case	Rate^#^		(95% CI)	Case	Rate^#^		(95% CI)
All	167	0.98	568	5.13	5.30(4.46, 6.29)***	5.38(4.52,6.40)***	161	3.94	4.10(3.30,5.09)***	4.05(3.26, 5.03)***
Gender										
Female	34	0.53	106	2.71	5.09(3.46, 7.48)***	5.33(3.61,7.86)***	48	2.62	4.94(3.18,7.66)***	4.40(2.83, 6.85)***
Men	133	1.24	462	6.46	5.27(4.35, 6.39)***	5.37(4.43,6.53)***	113	5.02	4.13(3.21,5.31)***	3.94(3.06,5.07)***
Age										
<65	33	0.44	163	2.96	6.77(4.66, 9.85)***	6.74(4.63,9.81)***	39	2.53	5.87(3.69, 9.33)***	6.00(3.76,9.56)***
65–74	77	1.32	268	8.02	6.18(4.79, 7.96)***	6.05(4.69,7.82)***	85	5.18	4.02(2.95, 5.47)***	4.29(3.15,5.86)***
≥75	57	1.53	137	6.20	4.12(3.02, 5.61)***	3.98(2.92,5.43)***	37	4.12	2.75(1.82,4.17)***	2.93(1.93,4.45)***
Urbanization level										
1 (highest)	34	0.72	125	4.55	6.38(4.37,9.32)***	6.70(4.58,9.81)***	41	4.09	5.81(3.68,9.16)***	5.21(3.30,8.24)***
2	42	0.94	162	5.43	5.78(4.11, 8.11)***	6.03(4.29,8.48)***	43	3.78	4.05(2.64, 6.19)***	3.89(2.54,5.96)***
3	33	1.12	96	5.08	4.62(3.11, 6.86)***	4.31(2.89,6.44)***	27	4.28	3.94(2.37, 6.56)***	3.59(2.15,6.00)***
4 (lowest)	58	1.17	185	5.37	4.64(3.45, 6.23)***	4.79(3.56,6.44)***	50	3.81	3.30(2.26, 4.83)***	3.74(2.56,5.47)***
Monthly Income(NTD^&^)										
<15,000	62	1.08	215	5.98	5.61(4.23,7.44)***	5.60(4.22,7.44)***	63	4.11	3.88(2.73,5.51)***	3.84(2.70,5.45)***
15,000–19,999	78	1.01	265	5.06	5.05(3.93,6.50)***	5.06(3.92,6.52)***	70	3.73	3.75(2.71,5.17)***	3.94(2.84,5.45)***
≥20,000	27	0.74	88	3.94	5.32(3.46,8.19)***	5.96(3.86,9.20)***	28	4.16	5.68(3.34,9.63)***	4.89(2.87, 8.33)***

Rate^#^, incidence rate of lung cancer, per 1000 person-years; ^&^NTD: New Taiwan dollar; Crude HR*, relative hazard ratio; Adjusted HR^†^: multiple analysis including age, sex, urbanization, monthly income(NTD) and co-morbidities of pneumoconiosis, interstitial lung disease, and pulmonary TB; ***model p<0.001.

Table 3 shows that the overall risk of lung cancer was 25% lower in the COPD with T2DM cohort than in the COPD without T2DM cohort (adjusted HR = 0.75, 95% CI = 0.63–0.90). The COPD without T2DM cohort was defined as the reference group, and the gender-specific risk of lung cancer in COPD patients with T2DM compared with those without T2DM was analyzed. The risk of lung cancer was significantly reduced in males (adjusted HR = 0.73, 95% CI = 0.60–0.90) In age-specific analysis, a reduced risk of lung cancer was observed for patients aged 65–74 years (adjusted HR = 0.70, 95% CI = 0.55–0.90). Furthermore, there was a significant reduction in the risk of lung cancer in subjects with an urbanization level of 2 or a monthly income <15,000 New Taiwan Dollars (adjusted HR = 0.64, 95% CI = 0.46–0.90 and adjusted HR = 0.69, 95% CI = 0.52–0.91, respectively).

**Table 3:** Hazard ratios of lung cancer between all COPD patients with and without T2DM stratified by demographic characteristics.

	T2DM	
	No	Yes
	Adjusted HR^†^ (95% CI)	Adjusted HR^†^ (95% CI)
All	1(Reference)	0.75(0.63, 0.90)**
Gender		
Women	1(Reference)	0.82(0.58, 1.16)
Men	1(Reference)	0.73(0.60, 0.90)**
Age		
<65	1(Reference)	0.89(0.63, 0.127)
65–74	1(Reference)	0.70(0.55, 0.90)**
≥75	1(Reference)	0.73(0.51, 1.06)
Urbanization level		
1 (highest)	1(Reference)	0.78(0.55, 1.12)
2	1(Reference)	0.64(0.46, 0.90)*
3	1(Reference)	0.84(0.54, 1.29)
4 (lowest)	1(Reference)	0.78(0.57, 1.07)
Monthly Income(NTD&)		
<15,000	1(Reference)	0.69(0.52, 0.91)**
15,000–19,999	1(Reference)	0.77(0.59, 1.01)
≥20,000	1(Reference)	0.82(0.53, 1.26)

& NTD: New Taiwan dollar; Adjusted HR^†^: multiple analysis including age, sex, urbanization, monthly income(NTD) and co-morbidities of pneumoconiosis, interstitial lung disease, and pulmonary TB; * model p<0.05, ** model p<0.01

## Discussion

To the best of our knowledge, this study, examining a large population cohort in Taiwan during a 14-year follow-up period is the first to investigate the risks of lung cancer in patients diagnosed with COPD either with or without T2DM. One key finding of this study was that, there was a significantly higher incidence of lung cancer among COPD patients (adjusted HR = 5.02, 95% CI = 4.23–5.94) compared to that of the general population. Further analyses indicated that there was a significantly protective effect from lung cancer (HR = 0.75, 95% CI = 0.63–0.90) of diabetic patients than non-diabetic patients among patients with COPD.

Several studies have shown increased risk of lung cancer in patients with COPD. Most of them concluded that COPD is an independent risk factor. However, the causal relationship between COPD and lung carcinogenesis is not yet fully understood. COPD can be exacerbated by pulmonary infections that cause inflammation, which contributes to carcinogenesis by generating reactive oxygen or nitrogen species, increasing cellular proliferation, upregulating antiapoptotic pathways, and stimulating angiogenesis [27]. Infection can also promote airway remodeling that enhance carcinogenesis [28]. Some studies have found that reduced lung function is an important feature in the development of lung cancer, and forced expiratory in one second (FEV_1_) has been thought to be an association between COPD and lung cancer [17].

T2DM is associated with hyperglycemia, hyperinsulinemia, insulin resistance, chronic inflammation, and oxidative stress. These conditions may induce abnormalities in cell physiology, cellular growth, regulation, and carcinogenesis [29]. For more than 50 years, clinicians have reported the occurrence of cancer in patients with concurrent diabetes. Subsequently, an association between diabetes and cancer has been identified in many population-based studies. More recently, the results of several studies have been combined for meta-analysis, indicating that some cancers such as liver, pancreas, endometrium, colon/rectum, breast, and bladder develop more commonly in patients with diabetes, predominantly in type 2. Until now, there has been sparse data evaluating the link with type 1 diabetes [22]. With regard to diabetes and lung cancer, the relationship between these two diseases remains inconclusive. Majority of previous studies have reported no increased risk of lung cancer in diabetic patients [30].

In our study, we found an interesting finding that patients with T2DM have a protective effect from getting lung cancer than those without T2DM among patients with COPD. Possible explanations for this include lifestyle modification of the patient (e.g., while a diabetic patient was diagnosed to have COPD, he/she may suffer more stress and be more motivated to make some changes). Smoking cessation is the most important behavior modification for COPD control. Meanwhile, this is also the most important key point to decrease the risk of developing of lung cancer. Second, T2DM itself is associated with less lung cancer incidence. Although there were less evidences and the mechanism was unknown, we still could find some studies support this hypothesis. Ogunleye *et al*. reported lung cancer incidence was 0.8% in diabetic patients and 1.0% in non-diabetic patients with an AHR was 0.77 (95% CI = 0.51–1.01) in a cohort study, which the total population was 9577 [31]. Atchison *et al.* observed the risk ratio of lung cancer in diabetic males than non-diabetic males was 0.8, 0.78, and 0.79 in 2–5, 6–10, and >10 years follow-up in a total 4,501,578 population [32]. Another large retrospective cohort study that used the same NHI database in Taiwan by Lo *et al.* revealed that AHR of lung cancer in diabetic patients than non-diabetic patients was 0.85 (95% CI = 0.81–0.89) and 0.92 (95% CI = 0.88–0.97) in <3.5 and >3.5 years follow-up with a total 1,790,868 population [33]. Some have hypothesized that a higher body mass index (BMI) found in patients with T2DM may decrease the risk of certain cancers such as esophageal and lung cancer. However, the exact reasons remain inconclusive. Third, medications for T2DM may have a protective role to prevent lung cancer. There is some evidence that a few kinds of anti-diabetic medications could reduce risk of cancer. Among the medications, most researchers were interested in the most commonly used anti-diabetic drug, metformin. Noto *et al.* reported a systemic review and meta-analyses that the use of metformin in diabetic patients was associated with significantly lower risks of cancer mortality and incidence [34]. Lai *et al.* reported anti-diabetic medications such as metformin, thiazolidinediones, and alpha-glucosidase inhibitors considerably decreased the risk of lung cancer [35]. However, Smiechowski *et al.* reported metformin use is not associated with a decreased risk of lung cancer in an UK database [36]. Finally, someone may consider that reduced life expectancy as a result of diabetes itself may decrease the incidence of lung cancer that occurs more frequently in later life. However, in our study, the mean following time of the diabetic cohort (7.01 years) was just a little shorter than that of the non-diabetic cohort (7.42 years).

The strengths of our study included its use of population-based data that are highly representative of the general population. However, certain limitations to our findings should be considered. First, the National Health Insurance Research Database (NHIRD) does not contain detailed information regarding smoking habits, diet preference, occupational exposure, drug history, and family history, all of which may be risk factors for lung cancer. Second, the evidence derived from a retrospective cohort study is generally lower in statistical quality than that from randomized trials because of potential bias related to adjustments for confounding variables. Despite our meticulous study design and attempt to control for confounding factors, bias resulting from unknown confounders may have affected our results. Third, all data in the NHIRD are anonymous. Thus, the relevant clinical variables, such as serum laboratory data, pulmonary function tests, imaging results, and pathology findings were unavailable for the patients in our study. Otherwise, the data regarding COPD, T2DM, and lung cancer diagnoses were nonetheless reliable. Last, although treatment (drug) effect may be critical for evaluating the association from T2DM to lung cancer. However, the NHIRD we used for this study does not contain anti-diabetic medication information in detail. It is difficult to perform the analysis. Nevertheless, this is a good idea for further research.

## Conclusion

Patients with COPD had a significantly higher risk of developing lung cancer than healthy people. However, there was a protective effect of T2DM for lung cancer among patients with COPD. Further investigation may be needed to corroborate the mechanism or bring up reliable reasons.
